# A new family of copper-based MXenes

**DOI:** 10.1038/s41598-021-90628-2

**Published:** 2021-06-11

**Authors:** R. Ponce-Pérez, S. J. Gutierrez-Ojeda, J. Guerrero-Sánchez, María G. Moreno-Armenta

**Affiliations:** Centro de Nanociencias y Nanotecnología, Universidad Nacional Autónoma de México, Km. 107, Apdo. 14 Carretera Tijuana-Ensenada, Ensenada, Baja California Mexico

**Keywords:** Materials science, Atomistic models

## Abstract

In this work, we demonstrate, through first-principles calculations, the existence of a new family of copper-based MXenes. These add up new structures to the previously reported universe and span the interest of such 2D materials for applications in heterogeneous catalysis, ion-based batteries, sensors, biomedical applications, and so on. First, we propose the MXene-like structures: Cu_2_N, Cu_2_C, and Cu_2_O. Phonon spectra calculations confirmed their dynamical stability by showing just positive frequencies all through the 2D Brillouin zone. The new MXenes family displays metallic characteristics, mainly induced by the Cu-3d orbitals. Bader charge analysis and charge density differences depict bonds with ionic character in which Cu is positively charged, and the non-metal atom gets an anionic character. Also, we investigate the functionalization of the proposed structures with Cl, F, O, and OH groups. Results show that the H3 site is the most favorable for functionalization. In all cases, the non-magnetic nature and metallic properties of the pristine MXenes remain. Our results lay the foundations for the experimental realization of a new MXenes family.

## Introduction

Atomic understanding of 2D systems is a critical factor to help in the nanoscale revolution. The hunt for 2D systems with engineered properties has been a constant since the graphene discovery in 2004^1^. To mention, silicene^2^, germanene^3^, phosphorene^4^, borophene^5^, and very recently, the plumbene monolayer^6^. All these systems with a great variety of properties and applications. Nevertheless, that is not it. Other binary and ternary 2D materials have emerged as well^7–9^, between them, 2D Janus^10^ and MXenes^11^. The later with several applications in different fields^12^. It is expected that the MXenes family could be one of the largest in the 2D realm. Since the outbreak work by Michael Naguib et al.^11^ on Ti_3_C_2_, several experimental and theoretical investigations have appeared on different MXenes. For example, antiferromagnetic Cr_2_N undergoes a half-metal transition just by adsorbing O on it, being applicable in spintronics^13^. Also, MXenes find applications in next-generation shielding^14^, nano-optoelectronics^15^, as key to efficient catalytic processes^16^, and in energy conversion and storage^17^. So, the spectrum of applications that pristine MXenes bring is still under construction and could be complemented with the addition of new MXene structures. In this way, several research groups are looking for new and novel MXenes with engineered properties through computational and experimental methods^18–26^. MXenes modification through surface functionalization is a possibility. For example, carboxyl functionalized Ti_3_C_2_T_x_ MXene shows a high capability to trap and remove U and Eu^20^. Also, sulfur-modified MXenes achieve high storage in Li-ion-based batteries^21^.

Mixing two transition metals into the same structure through the formation of either solid solutions or ordered double-transition metal MXenes is another way to engineer the MXenes properties. Ordered double-transition MXene sheets present an optically controlled ferrimagnetic to ferromagnetic transition^23^. Mixed functionalized double MXenes show an antiferromagnetic behavior useful to construct 2D-based spin field-effect transistors^26^. Also, double non-magnetic transition metal MXenes have been used as Li-storage anodes, in which surface storage is enhanced due to the ordered double-transition metal nature of the MXene layer^27^.

MXenes are commonly obtained from the precursor MAX phase through selective etching of the A layer, employing aqueous hydrofluoric acid solutions^28^. However, the selective atomic substitution method has been proposed as another way to obtain MXenes. Urbankowski et al.^29^ reported the synthesis of Mo_2_NT_x_ and V_2_NT_x_ using as precursors the Mo_2_CT_x_ and V_2_CT_x_ by ammoniation. Sun et al.^30^ and Cao et al.^31^ synthesized the two-dimensional Mo_5_N_6_ employing MoS_2_ layers as a precursor in a furnace with NH_3_ flux, and they demonstrated the versatility of this approach synthesizing W_5_N_6_. Besides, theoretical investigations have demonstrated that the substitution of M atoms in the MXene phase by other metallic atoms is possible^32,33^; for example, Ti_3_C_2_ has been modified by substituting Ti atoms with 3d, 4d, and 5d transition metals^32^. The MXenes MCO_2_ (M = Ti, V, Zr, Hf, and Ta) were modified by the replacement of the M atom by Cu, Zn, Mo, Ru, Rh, Pd, Ag, Cd, W, Re, Os, Ir, Pt, Au and Hg^33^. Taking into account that MXenes not only come from the MAX phases -they can also be synthesized- opens the door to include more members to the MXenes family.

Metallic copper has a wide range of applications; due to its high electromigration, it is widely used to construct integrated circuits. Also, Cu is an efficient current collector due to its high electric conduction^34^. Large area and conformal thin Cu films can be achieved on solid surfaces via atomic layer deposition, in which a Cu-based organometallic molecule interacts with the solid surface to generate the growth. The process happens through ligand-exchange reactions^35^. Also, copper surfaces can be used as a substrate to grow graphene, being a natural support to achieve graphene-based electrodes^34^.

That is not it; Cu is also widely used in single atom alloy catalysts^36^, in which single Pt atoms are incorporated into the Cu surface. Cu generates a high selectivity in hydrogenation processes due to its favored Cu–O interaction with the aldehyde molecules, whereas Pt is the hydrogen source.

We have great expectations of the potential advantages of bringing a 2D material with two exposed Cu surface layers and a large surface area. For example, due to its affinity to graphene, it is natural to think in new vdW heterostructures, which will be highly interesting from the scientific point of view as well as for their potential applications. On the other hand, the feasibility of mixing MXenes with other transition metals assures the formation of single-atom alloy catalysts, in which Pt, Pd, or other relatives can be incorporated. Several other applications related to copper should be expected as well.

Considering the previous discussion, we propose a new copper-based MXenes family: Cu_2_C, Cu_2_N, and Cu_2_O, which are isostructural to the well-known and already studied Cr_2_C, Cr_2_N, Ti_2_C, Ti_2_N structures^13,32,37,38^. Our results demonstrate that the new MXenes family presents positive frequencies and metallic behavior, even after functionalizing them with Cl, F, O, and OH, a key characteristic that remains from pure copper, pointing out the importance of such 2D structures experimental realization.

## Methodology

Calculations on this report were performed using the Vienna Ab initio Simulation Package (VASP)^39–42^. Exchange–correlation energies are treated according to the generalized gradient approximation (GGA) with the Perdew-Burke-Ernzerhof (PBE) parameterization^43^. The electronic states are expanded using the projector-augmented wave basis (PAW)^44,45^ with an energy cutoff of 500 eV. The MXene-type structures Cu_2_X (X = O, N, C) are simulated with the supercell method. A monolayer of Cu_2_X composes each supercell in a 1 × 1 periodicity and a vacuum space of 15 Å to avoid spurious interactions. In geometry optimization, all force components must be lower than 0.01 eV/ Å and the energy differences less than 1 × 10^–4^ eV. Integration of electronic states in the Brillouin zone was performed using a Monkhorst–Pack mesh of 15 × 15 × 1 k-points^46^. Phonon dispersions were obtained using the finite-differences method^47^ combined with phonopy code^48^.

## Results

**Figure 1:**
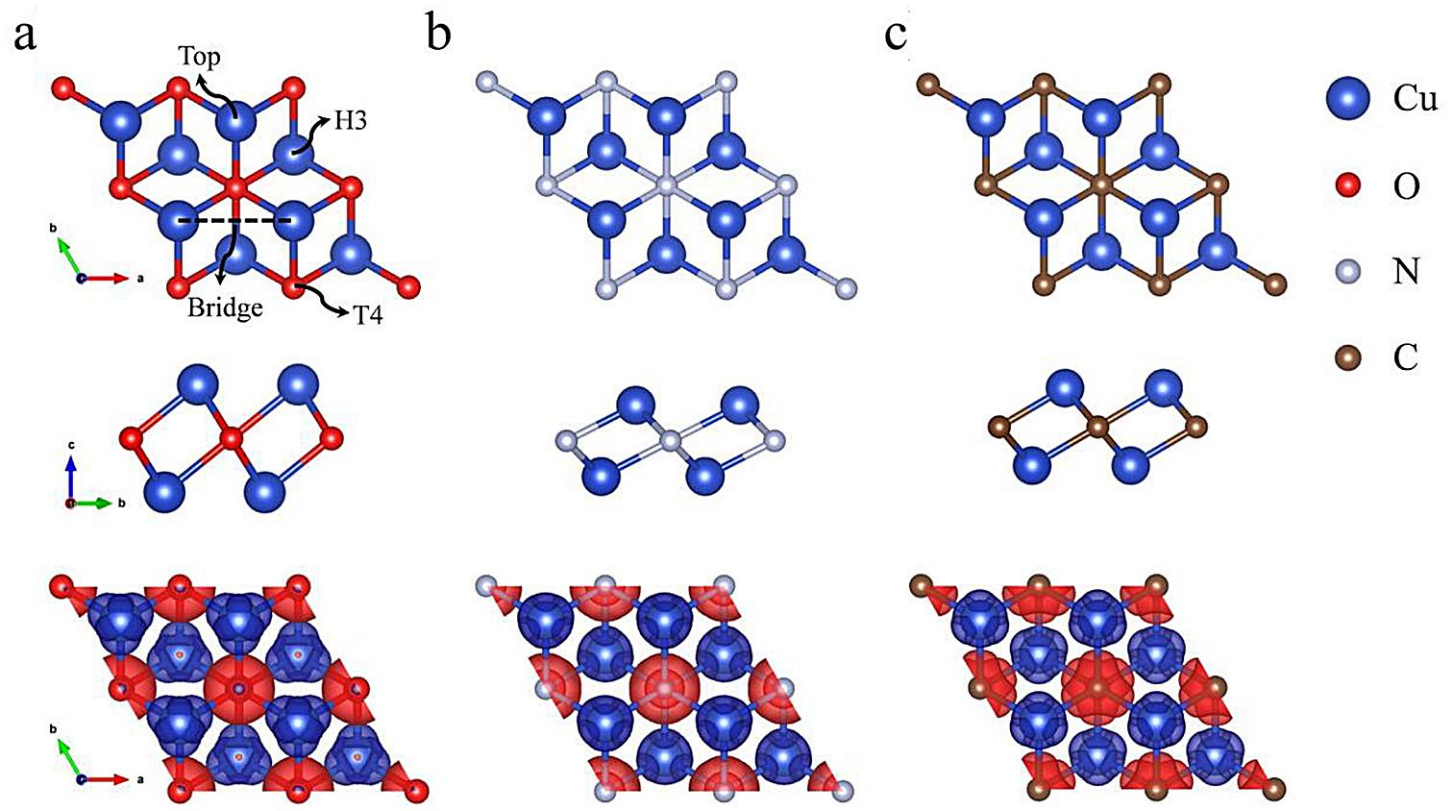
Atomistic representation of the new Cu-based MXene-type structures, the top and side views of (**a**) Cu_2_O, (**b**) Cu_2_N, and (**c**) Cu_2_C. The bottom part represents the electron density difference (Δ⍴). Blue represents depletion, and red is for the accumulation of charge.

The atomic representation of the new Cu-based MXenes is depicted in Fig. 1. Top and side views of the Cu_2_O, Cu_2_N, and Cu_2_C are shown in Fig. 1a–c, respectively. After full structural optimization without any constraints, the Cu_2_O, Cu_2_N, and Cu_2_C have cell parameters of the order of 2.80, 3.12, and 2.99 Å, respectively. The Cu–O, Cu–N, and Cu–C bonds have distances ~ 2.07, ~ 2.02, and ~ 2.00 Å. Each MXene-type structure comprises two monolayers (ML) of Cu as the most exposed and a central ML of X (X = O, N, C). The Cu-X interlayer distance is 1.30, 0.92, and 1.02 Å for the Cu_2_O, Cu_2_N, and Cu_2_C, respectively.

We calculated the electron density difference (Δ⍴) defined as $\Delta \rho = \rho_{MXene} - \rho_{{{\text{Cu}}}} - \rho_{X}$, where ⍴_MXene_ is the charge density of the MXene-type structure; ⍴_Cu_ and ⍴_X_ are the charge density of the isolated atoms of Cu and X (X = O, N, C) respectively. The isosurfaces for each Cu-based MXene are depicted in Fig. 1, blue color suggests depletion, and the red color corresponds to charge density accumulation. The three different Cu-based MXenes denote charge depletion around the Cu atoms. Also, we observed the accumulation of charge close to the Oxygen atoms. This behavior suggests bonds with ionic behavior. Bader charge density analysis for the Cu_2_O MXene-type structure reveals that each Cu atom transfers 0.50e to the three nearest O atoms (0.166e/O); also, each O atom accepts 1.00e from the six Cu nearest neighbors. In the Cu_2_N MXene-type structure, Cu atoms donate 0.60e (0.20e/O atom) to the nearest N atoms, which accept 1.20e from the six Cu nearest neighbors. Similar behavior is observed for the Cu_2_C MX-type structure, where Cu atoms lose 0.48e (0.16e/O atom) and the O atoms accept 0.96e from the six nearest Cu atoms.

**Figure 2:**
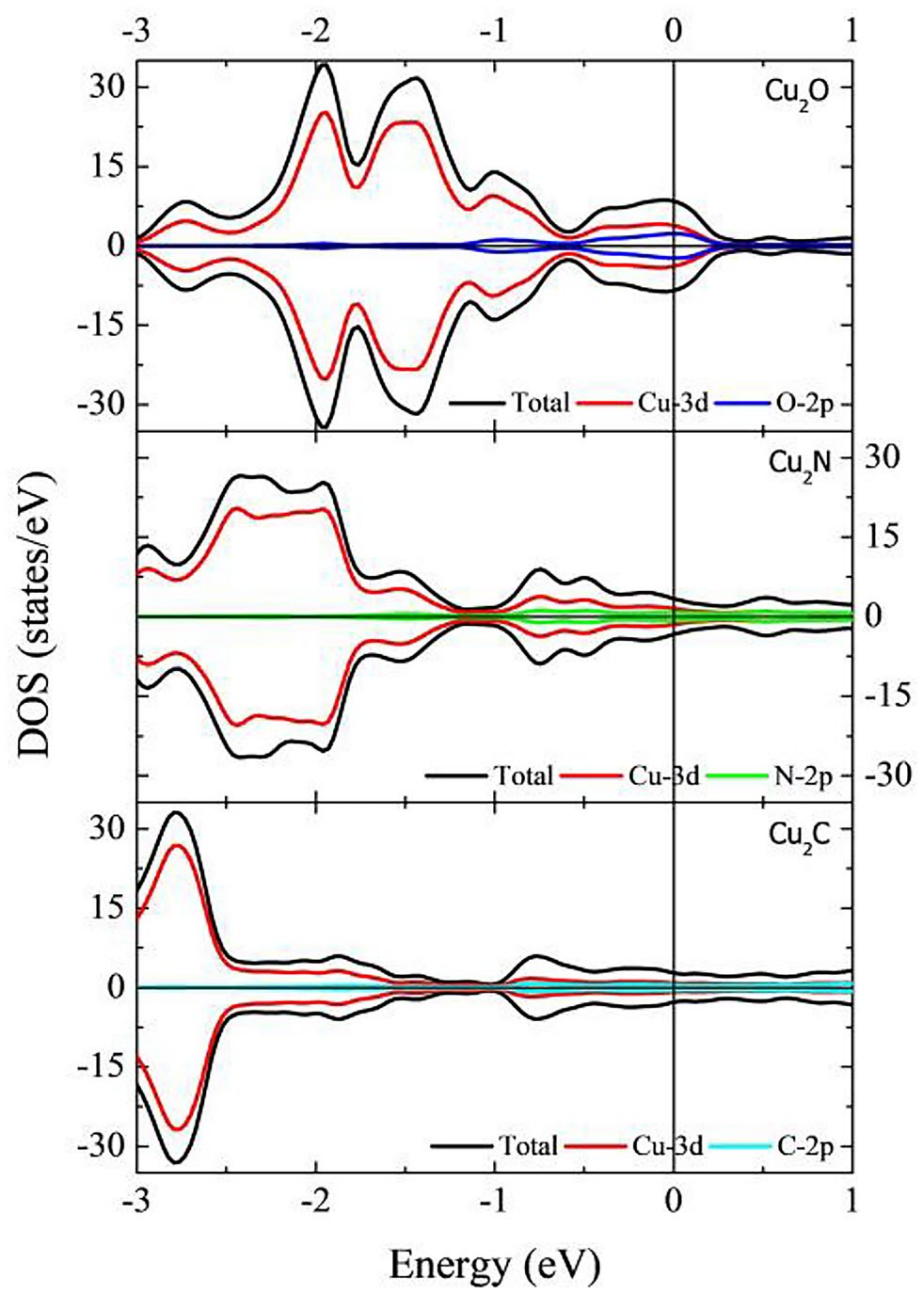
DOS and pDOS for the Cu_2_O, Cu_2_N and Cu_2_C MXenes. Fermi energy is set to 0 eV.

The electronic properties of the new Cu-based MXene-type structures are investigated. The results are summarized in Fig. 2. The density of states (DOS) and projected DOS (pDOS) for the Cu_2_O, Cu_2_N, and Cu_2_C are shown in the upper, middle, and lower panels of Fig. 2, respectively. Fermi level is set as the reference energy in all cases. Positive and negative values along the DOS axis are for spin up and spin down, respectively. In all cases, spin up and spin down show no asymmetry as a clear indication of the material's non-magnetic nature. Also, all the Cu-based MXenes present metallic characteristics. Cu-3d orbitals dominate DOS in all cases, from − 3 to 1 eV, with small contributions coming from the O-2p, N-2p, and C-2p orbitals.

**Figure 3:**
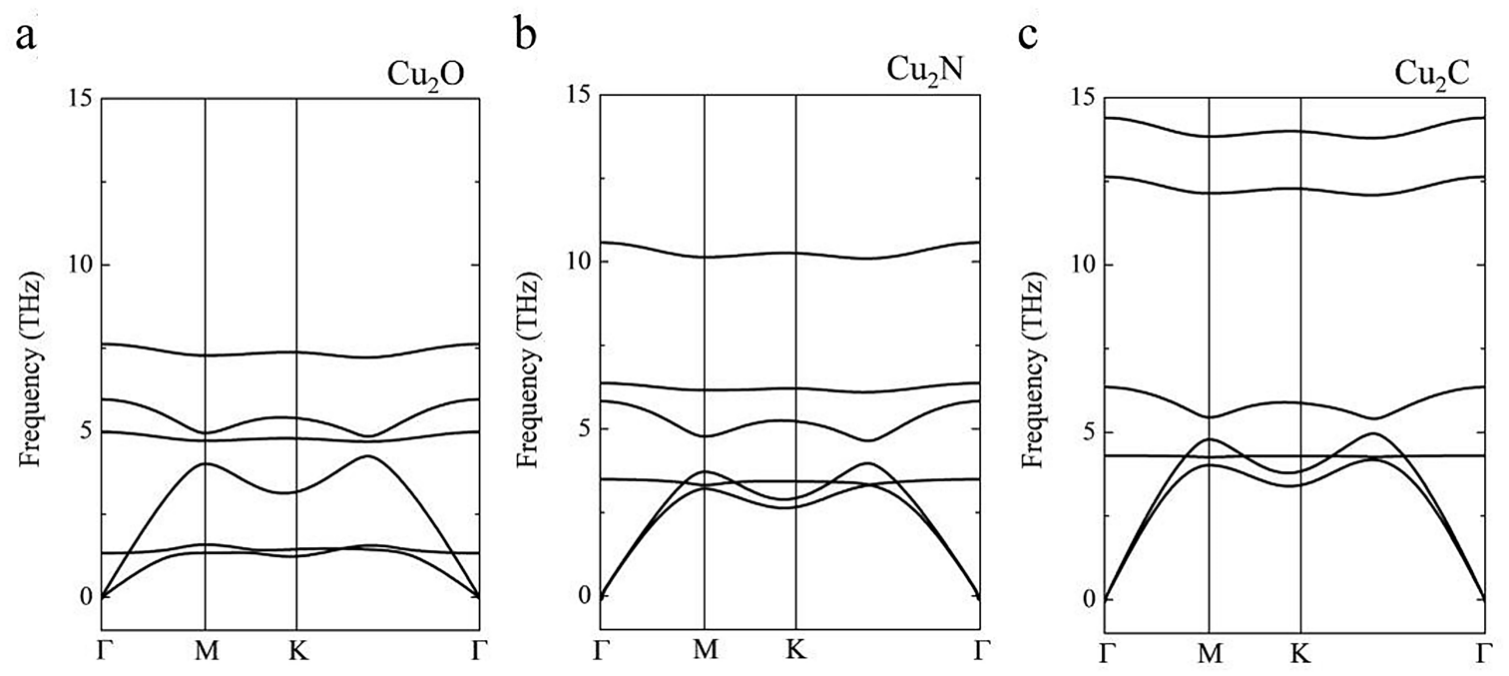
Phonon dispersion for the Cu-based MXene (**a**) Cu_2_O, (**b**) Cu_2_N, and (**c**) Cu_2_C.

The evidence that demonstrates the possible experimental realization of the Cu-based MXenes is the dynamic stability. In the phonon dispersion, positive frequencies stand for dynamically stable systems, whereas negative values suggest structural instabilities that may need to be corrected using strain or atomic displacements. Here we analyze the phonon dispersions of the new Cu-based MXenes along the path Γ–M–K–Γ (Fig. 3). Dispersions for Cu_2_O, Cu_2_N, and Cu_2_C are shown in Fig. 3a–c, respectively. In all cases, the absence of negative frequencies indicates dynamical stability of the three different Cu-based MXenes.

MXenes are generally obtained with some functional groups attached to them. The most common functional groups are Cl, F, O, and OH. We investigate the surface functionalization of the three different Cu-based MXenes with Cl, F, O, and OH to investigate the change in the electronic properties when functionalizing them.

**Figure 4:**
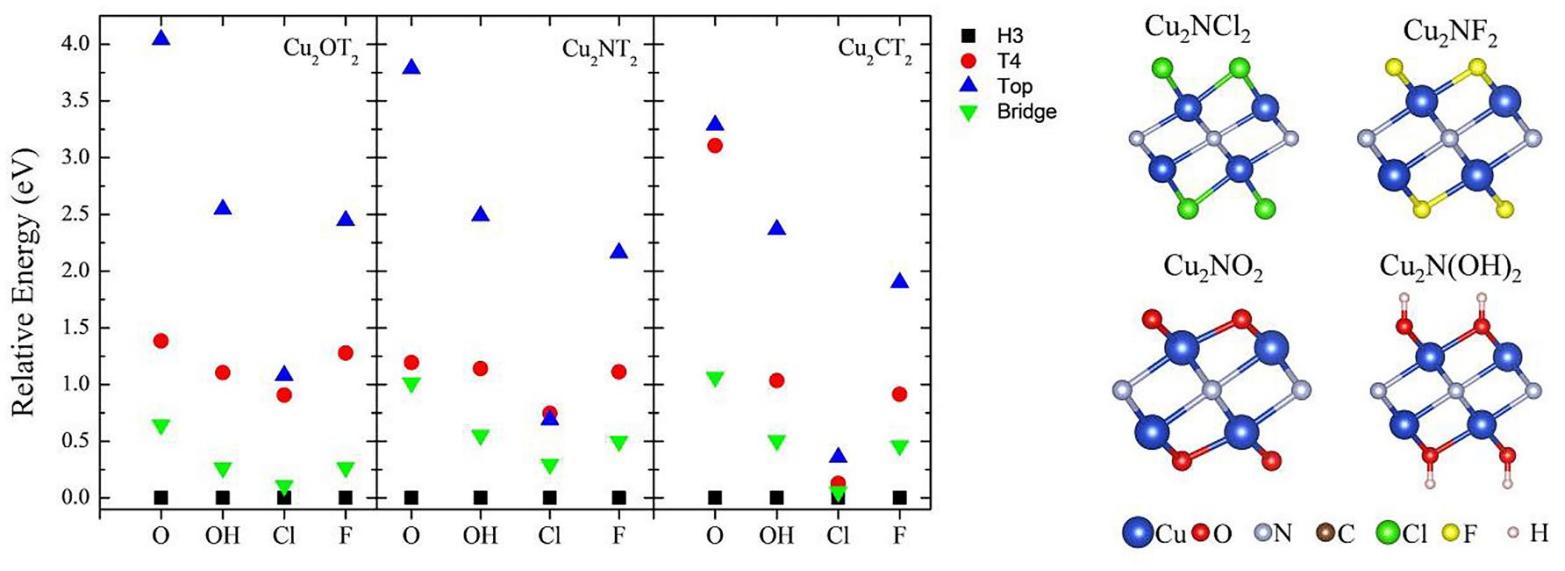
Relative energies for the different Cu_2_XT_2_ (X = O, C, N and T = Cl, F, O, OH), the atomistic model for the Cu_2_NT_2_ are shown.

To evaluate the most favorable site for the functionalization, we considered four high symmetry sites (see Fig. 1a). The Top site corresponds to the site on top of the Cu atom. The T4 site is on top of the central layer atom, H3 is the central point of the hexagon formed by the 1st and second layers, and the Bridge site is the middle point between two Cu atoms of the same layer. According to the calculations, the H3 site is the most favorable configuration in all cases. In general, the H3 site is the most stable functionalization site for all systems, followed by the bridge site. T4 and Top sites are the least stable (H3 < Bridge < T4 < Top). Relative energies of the different functionalized MXenes are summarized in Fig. 4.

After structural relaxation, the functional groups are threefold coordinated to the Cu atoms. In the case of Cl, the average bond distances with the Cu atoms are 2.36 Å, 2.37 Å, and 2.41 Å, and the interplanar distances are 1.39 Å, 1.45 Å, and 1.48 Å for the Cu_2_OCl_2_, Cu_2_NCl_2_, and Cu_2_CCl_2_, respectively. For functionalization with F atoms, the three different MXenes exhibit the same bond distance (2.04 Å). The interplanar distances are 0.97 Å, 1.07 Å, and 1.10 Å for the Cu_2_OF_2_, Cu_2_NF_2_, and Cu_2_CF_2_, respectively. Similar results are obtained for the Cu_2_O_3_, Cu_2_NO_2_, and Cu_2_CO_2_ MXenes. The Cu–O bond distances are 1.90 Å, 1.91 Å, and 1.94 Å, and the interplanar distances 0.82 Å, 0.83 Å, and 0.78 Å, respectively. Finally, the MXene group functionalized with OH has Cu–O bond distances of the order of 2.07 Å, 2.06 Å, and 2.08 Å. Also, the interplanar distances are 0.99 Å, 1.05 Å, and 1.08 Å for the Cu_2_O(OH)_2_, Cu_2_N(OH)_2_, and Cu_2_C(OH)_2_ MXenes, respectively. The atomistic models for the Cu_2_NT_2_ (T = Cl, F, O, and OH) are displayed in Fig. 4.

**Figure 5:**
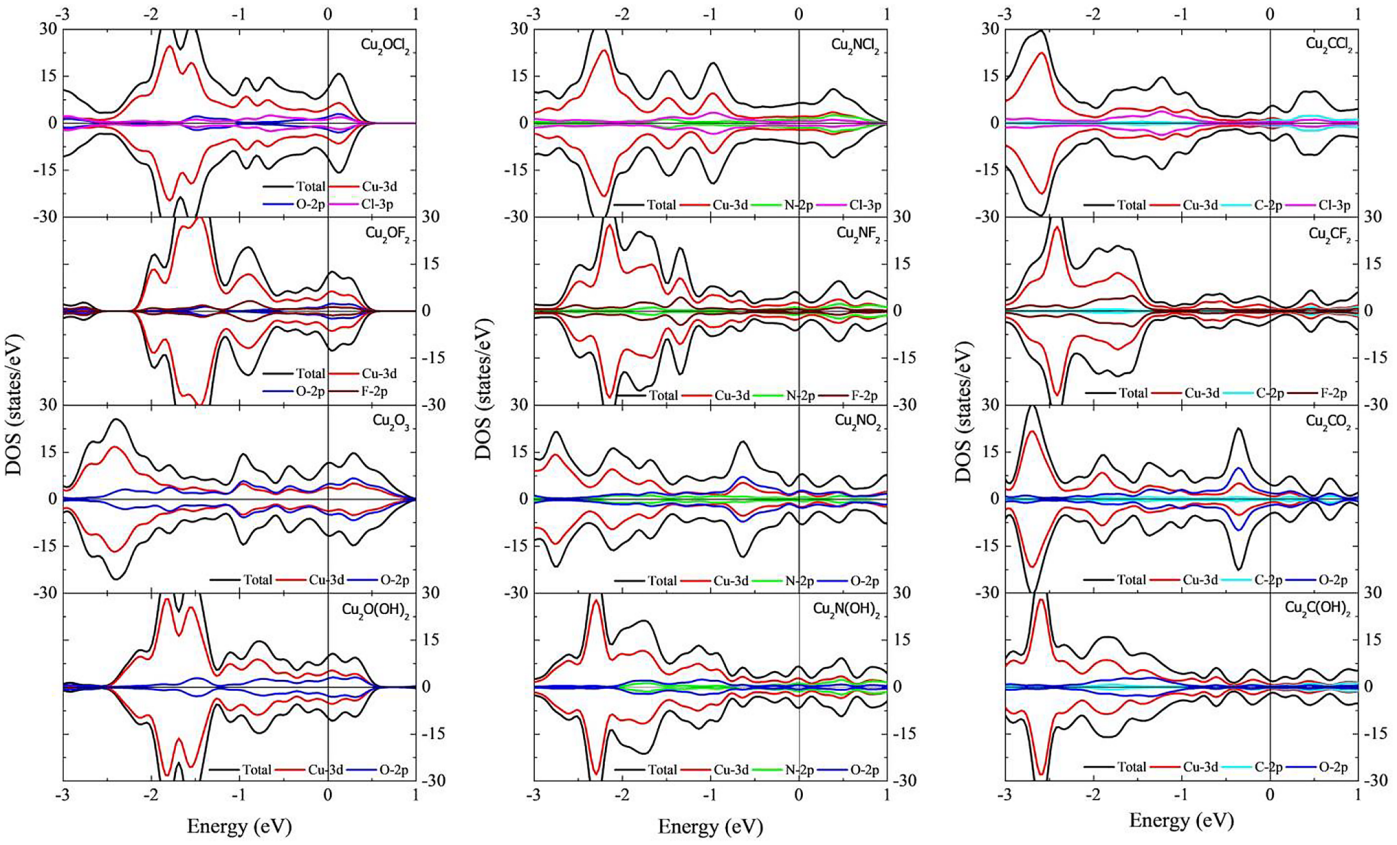
DOS and pDOS of the functionalized Cu_2_OT_2_, Cu_2_NT_2_ and Cu_2_CT_2_ (T = Cl, F, O, and OH) MXenes.

To evaluate the change in electronic properties in the Cu_2_XT_2_ family due to functionalization, we compute its electronic properties. The DOS and pDOS of the functionalized MXnes are summarized in Fig. 5. In all cases, the energy reference is the Fermi level, positive and negative values along the DOS axis refer to spin up and spin down, respectively. From left to right, the first column depicts the electronic properties of the Cu_2_OT_2_ (T = Cl, F, O, and OH) functionalized MXenes. Note that in all cases, the non-magnetic nature of the pristine MXene remains. Also, the main contribution to the DOS around the Fermi level comes from the Cu-3d orbitals, followed by contributions of the O-2p orbitals and the T group p-orbitals. Note that the surface functionalization increases the electronic states close to the Fermi level, and therefore the MXenes remain metallic. Similar behavior is obtained for the Cu_2_NT_2_ (T = Cl, F, O, and OH) functionalized MXenes (central column). Again, functionalized MXenes exhibit metallic characteristics with non-magnetic properties. DOS close to the Fermi is mainly composed of Cu-3d orbitals with a small N-2p and T-p orbitals contribution. The right column depicts the DOS and pDOS of the Cu_2_CT_2_ (T = Cl, F, O, and OH) functionalized MXenes. Similar to the previous cases, the non-magnetic nature and the metallic behavior of the pristine Cu_2_C MXene remain. Around the Fermi level, the main contribution to the DOS comes from the Cu-3d orbitals. C-2p and T-p orbitals contribute to a lesser extent.

## Conclusions

In conclusion, by using spin-polarized first-principles total-energy calculations, we predicted the structural and electronic properties of the new Cu-based MXenes Cu_2_O, Cu_2_N, and Cu_2_C. Calculations demonstrate the non-magnetic nature of the MXenes, with metallic characteristics. Functionalization of the proposed MXene structures is carried out employing Cl, F, O and OH groups. Computations show tha all functionalized species are metallic and non-magnetic, similar to the case of pristine MXenes. Also, phonon dispersion with only positive frequencies indicates the dynamical stability of the Cu-based MXenes. Therefore, this new MXenes family could be obtained experimentally, opening an avenue to include the large number of applications that Cu can bring to the realm of 2D materials.
